# Dietary supplementation with fermented plant product modulates production performance, egg quality, intestinal mucosal barrier, and cecal microbiota in laying hens

**DOI:** 10.3389/fmicb.2022.955115

**Published:** 2022-09-30

**Authors:** Yong Tian, Guoqin Li, Shuo Zhang, Tao Zeng, Li Chen, Zhengrong Tao, Lizhi Lu

**Affiliations:** ^1^State Key Laboratory for Managing Biotic and Chemical Threats to the Quality and Safety of Agro-Products, Institute of Animal Science & Veterinary, Zhejiang Academy of Agricultural Science, Hangzhou, China; ^2^Key Laboratory of Livestock and Poultry Resources (Poultry) Evaluation and Utilization, Ministry of Agriculture and Rural Affairs of China, Hangzhou, China

**Keywords:** fermented plant product, production performance, intestinal barrier, cecal microbiota, laying hens

## Abstract

Fermented plant product (FPP) is a kind of functional complex containing probiotics and a variety of bioactive substances, which has multiple physiological functions. However, there is no systematic appraisal of FPP as a feed additive for laying hens. This study was conducted to evaluate the utilization of FPP in laying hens. A total of 120 healthy 34-week-old Xianju layers with similar body weight and egg production were randomly allocated into two dietary treatments with four replicates per treatment and 15 birds per replicate for 8 weeks. The dietary treatments included the basal diet without FPP (CON group) and CON diet supplemented with 500 mg/kg of FPP (FPP group). Compared with the CON group, the egg production and egg mass were significantly increased in the FPP group from 38 to 42 and 34 to 42 weeks of age (*P* < 0.05). Birds fed with the diet containing 500 mg/kg FPP had higher albumen height (*P* < 0.01) and Haugh unit (*P* < 0.05) than those of the controls. FPP supplementation significantly increased the villus height (VH) and crypt depth (CD) in the jejunum of laying hens (*P* < 0.01), as well as the ratio of VH to CD (*P* < 0.05). The mRNA expression of tight junctions showed that dietary supplementation with FPP significantly increased the expression levels of *Occludin* (*P* < 0.01) and *ZO-1* (*P* < 0.05) in jejunum of hens compared to the control group. In addition, dietary supplementation with FPP influenced cecal microbiota of laying hens, which was characterized by the changes in the microbial community composition, including the increased abundances of *Firmicutes, Faecalibacterium, Oscillospira, Clostridium, Ruminococcus*, and *Coprococcus*, along with the decreased abundance of *Bacteroidetes, Proteobacteria, Phascolarctobacterium, Odoribacter, Desulfovibrio*, and *Mucispirillum*. Spearman's correlation analysis revealed that bacteria such as *Faecalibacterium, Ruminococcus, Coprococcus*, and *Blautia* were significantly and positively correlated with the intestinal barrier markers (*P* < 0.05), with extremely significant correlations between *Ruminococcus* and *ZO-1*, and *Coprococcus* and *Occludin* (*P* < 0.01), whereas *Desulfovibrio* had a negative correlation with the expression of *Occludin* (*P* < 0.05). As it can be concluded, FPP supplementation increased the egg production, egg mass, albumen height, and Haugh unit of laying hens, and improved intestinal health by ameliorating intestinal barrier function, which may be partially attributed to the regulation of cecal microbiota. Our findings suggest that FPP has the potential to be used as a feed additive to promote the performance of layers.

## Introduction

During the past few decades, antibiotics such as gentamicin, kanamycin, tetracycline, streptomycin, amoxicillin, penicillin, and bacitracin were used routinely as a poultry production performance promoters and as a preventive antimicrobial drug (Diarra and Malouin, 2014; Roth et al., 2019). However, the excessive and abusive use of antibiotics in the livestock industry led to antimicrobial resistance, serious adverse drug reactions, and antibiotic residues, which have become one of the potential threats to human health (Stanton, 2013; Hosain et al., 2021). With the constant increase in consumer demand for improved food safety, a change to the way of antibiotic use in the livestock industry has been caused. The Ministry of Agriculture and Rural Affairs of China has announced a ban on the production, import, operation, and use of growth-promoting pharmaceutical feed additives except for traditional Chinese medicine from 1 July 2020. As the use of antibiotics has been banned or reduced, antibiotic-free breeding is a hot issue in the development of animal husbandry, and it is urgent to find safe and effective antibiotic substitutes. Many studies have been conducted with the aim of improving animal productivity and immunity by feeding with various natural additives as potential alternatives to antibiotics (Cheng et al., 2014; Arowolo and He, 2018).

Fermented plant product (FPP) is a functional product fermented by various probiotics such as *lactobacillus*, yeasts, and molds based on one or more fresh vegetables, fruits, crops, mushrooms, marine algae, Chinese herbal medicines, and other plant-based raw materials, which contains a variety of biological enzymes, probiotics, prebiotics, polyphenols, secondary metabolites, short-chain fatty acids, vitamins, organic acids, minerals and other functional components (Feng et al., 2017). With numerous bioactive compounds, FPP plays essential roles in various physiological processes, including antioxidant, antibacterial, anti-inflammatory, enhancing immunity, improving intestinal barrier function, and modulating gut microbiota (Kim et al., 2019; Yudiarti et al., 2019; Cheng et al., 2020; Zhong et al., 2020). In addition, plant fermentation products provide health (Jayachandran and Xu, 2019) and beauty benefits (Lee et al., 2015).

As a modern functional fermentation product, FPP is produced by various microorganisms and contains several bioactive substances, which have many benefits as shown in humans and mice (Septembre-Malaterre et al., 2018; Yan et al., 2019). It is very popular in Japan, China, and some other Asian countries (or regions), and has been widely studied in the field of medical beauty. However, the research on FPP in poultry production is still at an initial stage, and its role and mechanism in poultry remain unclear. In the past few years, several studies have reported that diets supplemented with plant fermentation products can promote animal health and performance by improving antioxidant status, enhancing immune response, and regulating the homeostasis of intestinal flora (Li et al., 2019; Shahbazi et al., 2021; Tanaka et al., 2021). Although some evidence for FPP as a dietary additive has been reported in mammals, there are still limited data available, especially with regard to the effects of FPP on layers. The current study was, therefore, conducted to investigate the possible effects of FPP on the production performance, egg quality, intestinal morphology, cecal microbial profile, and gut barrier function in laying hens.

## Materials and methods

### Animals, treatments, management, and sample collection

A total of 120 healthy Xianju laying hens (34-week age) with similar body weight and egg production were used in this experiment. The hens were randomly allocated into one of the two dietary treatment groups, with four replicates per treatment and 15 birds per replicate. Dietary treatments included a control basal diet without additive (CON), as well as the basal diet supplemented with 500 mg/kg of the fermented plant product (FPP). FPP used in this study was a powdered product made from *Lactobacillus acidophilus* and *Bacillus subtilis* as fermenting strains and Chinese medicinal herbs as raw materials, after liquid fermentation, ultrasonic wall breaking, and extraction of active ingredients. The additive functional plant fermentation product was provided by Zhejiang Kangwan Tokugawa Technology Co., Ltd. (Shaoxing, Zhejiang province, China). Before commencing the experiment, all birds were fed with a commercial layer diet (basal diet), and the composition and nutrient levels of the commercial basal diet are shown in Table 1.

**Table 1:** Ingredients and nutrient levels of basal diet for the laying hens.

**Items**	**Content**
Ingredients (%)	
Corn	55.00
Soybean meal	25.00
Rice bran	4.00
Fish meal	3.00
Oyster shell	3.00
Tricalcium phosphate	6.50
Monocalcium phosphate	1.20
Salt	0.30
Premix	2.00
Total	100.00
Nutrient levels	
Metabolizable energy (MJ/kg)	11.30
Crude protein (%)	17.00
Calcium (%)	3.50
Phosphorus (%)	0.52
Lysine (%)	0.80
Methionine + cystine (%)	0.65

Premix provided as following (per kilogram of diet): vitamin A, 8,000 IU; vitamin D_3_, 2,200 IU; vitamin E, 10 IU; vitamin K_3_, 1.5 mg; vitamin B_1_, 1.5 mg; vitamin B_2_, 3.5 mg; vitamin B_6_, 3 mg; vitamin B_12_, 0.1 mg; nicotinic acid, 30 mg; pantothenic acid, 8 mg; folic acid, 1 mg; Cu, 8 mg; Fe, 80 mg; Mn, 60 mg; Zn, 40 mg; I, 0.18 mg; Se, 0.3 mg. The levels of crude protein, calcium, phosphorus, lysine, and methionine + cystine were measured values, while metabolizable energy level was obtained by calculation.

The hens were housed in two-tier battery cages (39 × 40 × 37 cm; length × width × height) with three hens per cage, and each cage was equipped with a feed trough and nipple drinker. Diets and water were available *ad libitum*. All birds were raised in an environmentally controlled facility throughout the experimental period of 8 weeks (until 42 weeks of age). The room temperature was maintained at 21 ± 2°C, and the relative humidity was approximately 65%. During the experiment, the hens have received no vaccinations and given artificial light by a daily lighting schedule of 16 h light and 8 h dark (16L: 8D).

### Laying performance and egg quality

Eggs were collected and weighed daily throughout the feeding trial. The data of egg number and weights were used to calculate egg mass, and egg production percentage was expressed as an average hen-day production based on the number of days at 4-week intervals. Feed intake was recorded daily on a replication basis (15 birds), and average daily feed intake per hen was calculated based on total feed intake and the number of days in the analyzed period. Feed conversion ratio (FCR) was calculated based on feed intake and egg mass.

A total of 40 saleable eggs (no shell defects, cracks, or double yolks) were randomly selected from each group and used for egg quality assessment at the end of the feeding experiment. The eggs were weighted individually. Eggshell breaking strength was determined using the eggshell force gauge (EFG-0503, Robotmation Co., Ltd., Tokyo, Japan). Eggshell thickness was measured on the large end, equatorial region, and small end, respectively, using an eggshell thickness gauge (EGT-1601, Robotmation Co., Ltd., Tokyo, Japan). Egg yolk color, albumen height, and Haugh unit were measured using an egg multi-tester (EMT-5200, Robotmation Co., Ltd., Tokyo, Japan).

### Morphometric examination of jejunal tissue

Two birds per replicate were randomly selected and euthanized by severing the jugular veins at the end of the feeding trial. The small intestines were disemboweled and approximately 5 cm segments from the middle part of the jejunum were excised. The collected intestinal segments were rinsed with ice-cold phosphate-buffered saline (PBS), fixed in 4% paraformaldehyde solution, dehydrated, and embedded in paraffin. Numerous sections with a thickness of 4–5 μm were cut, put on glass slides, stained with hematoxylin and eosin (HE), and used for histomorphological measurements under a light microscope. The measured parameters included villus height and crypt depth, and the ratio of villus height to crypt depth (VH/CD) was calculated. Intestinal villus height was measured from the apex of the villus to the villus-crypt junction, while crypt depth was measured from the base of the crypt up to the villus-crypt junction. The measurement values of villus height and crypt depth were obtained by using Image-pro plus 6.0 software (Media Cybernetics, Inc., Rockville, MD, USA).

### Gene expressions of tight junction components

The jejunal tissues from the slaughtered birds (two birds/replicate) were rinsed with ice-cold PBS. Intestinal mucosa was scraped off and immediately frozen in liquid nitrogen, then stored at −80°C for further analysis. The total RNA was isolated from each sample using TRIzol reagent (Invitrogen, Thermo Fisher Scientific, Braunschweig, Germany) in accordance with the manufacturer's procedures. Total RNA was reverse transcribed into cDNA for qPCR using HiScript II Q RT SuperMix (Vazyme Biotech, Nanjing, China) according to the manufacturer's protocol.

Real-time PCR assays were performed to determine expression using ChamQ Universal SYBR qPCR Master Mix (Vazyme Biotech, Nanjing, China) in a Roche LightCycler 96 system (Roche, Switzerland). Chicken *Occludin, ZO-1*, and *Claudin-1* genes were amplified using gene-specific primer pairs (Table 2), and β*-acting* served as an internal reference gene for normalization. The qPCR was performed in a 20 μl reaction mixture containing 10 μl of 2 × ChamQ Universal SYBR qPCR Master Mix, 1.2 μl of template cDNA, 0.4 μl of each primer (10 μM), and 8 μl of ddH_2_O. The reaction mixtures were incubated in a 96-well plate at 95°C for 1 min, followed by 40 cycles of 95°C for 15 s and 60°C for 30 s. At the end of the amplification cycle, a melting curve analysis was performed to confirm the specificity of the amplification. All reactions were carried out in three biological replicates. The relative expression levels of target genes were calculated using the 2^−ΔΔ*Ct*^ method. The ΔΔCt value represents the difference between the mean ΔCt of the FPP group and the CON group, where ΔCt indicates the difference between the mean Ct of the target gene and the internal reference gene for each sample.

**Table 2:** Primer sequences of chicken *Occludin, ZO-1, Claudin-1*, and β*-actin* genes used for RT-qPCR.

**Genes**	**Primer sequences 5^′^−3^′^**	**GenBank**
*Occludin*	F: GCTGAGATGGACAGCATCAA R: CCTCTGCCACATCCTGGTAT	NM-205128
*ZO-1*	F: TAAGGGGAAGCCAACTGATG R: GAAGGAGCAGGAGGAGGAGT	XM-413773
*Claudin-1*	F: CATGAAGTGCATGGAGGATG R: GTGCTGACAGACCTGCAATG	NM-001013611
*β-actin*	F: TCGCACTGGATTTCGAGCA R: CACCTGAACCTCTCATTGCCA	NM-205518

### Analysis of gut microbiome

As already pointed out, two birds per replicate were slaughtered for sampling at the end of the feeding trial. The cecal content from each bird was collected and immediately transferred into liquid nitrogen, then stored at −80°C. The cecal microbiota diversity of each sample was determined by high-throughput sequencing of 16S rRNA genes. To analyze the complete bacterial community composition, the V3–V4 hypervariable region of 16S rRNA genes was amplified using the barcoded primers (338F, 5′-ACTCCTACGGGAGGCAGCA-3′; 806R, 5'-GGACTACHVGGGTWTCTAAT-3′). PCR assays were performed using an Applied Biosystems 2,720 Thermal Cycler (Life Technologies, Carlsbad, CA, USA). The PCR products were subjected to 1.5% agarose gel electrophoresis and purified using the AxyPrep DNA Gel Extraction Kit (Axygen Biosciences, Union City, CA, USA) according to the manufacturer's recommendations. Pair-end libraries were generated following Illumina's genomic DNA library preparation procedure, and the library sequencing was performed using the Illumina NovaSeq 6000 platform (Illumina Inc., San Diego, CA). The original sequencing data have been deposited in the National Center for Biotechnology Information (NCBI) Sequence Read Archive (SRA) database under accession number PRJNA839201.

All reads were processed and analyzed with QIIME2 (v2019.4), Cutadapt (v2.3), and Vsearch (v2.13.4_linux_x86_64) software packages, and the clean data were then clustered as operational taxonomic units (OTUs) with a 97% similarity threshold. There was an obvious outlier in each group that might interfere with the statistical analysis of the microbiome and was therefore excluded from the subsequent analysis. Alpha diversity (Chao1, observed species, Shannon, and Simpson) of each sample was analyzed by QIIME2 software. Beta diversity was estimated by calculating Bray–Curtis distance, then visualized with principal coordinate analysis (PCoA) and non-metric multidimensional scaling (NMDS). To estimate the microbial community of the samples, the taxonomic assignment of OTU representative sequences was performed by the QIIME2 program based on the Greengenes 13.8 database. Heatmap of bacterial abundance was made using the R package (pheatmap). Linear discriminant analysis (LDA) effect size (LEfSe) was performed to determine the differences in bacterial taxonomy between the CON group and the FPP group (Segata et al., 2011).

### Statistical analysis

Data regarding production performance, egg quality, intestinal morphology, relative mRNA expression, and *Firmicutes* to *Bacteroidetes* ratio between two treatments in this study were statistically analyzed by independent sample *t*-test with SPSS 26.0 (SPSS Inc., Chicago, IL, USA). The results were presented as means and standard error of the mean (SEM). The levels of statistical significance were indicated as ^*^P < 0.05 and ^**^P < 0.01.

The Spearman's correlation analysis was performed to evaluate the potential links among jejunal barrier-related genes *Occludin, ZO-1*, and *Claudin-1*, and intestinal microbiota in laying hens. Data obtained from each sample (based on the 16S rRNA assay results) including mRNA expression of tight junctions and relative abundance of key members selected from the top 35 bacteria genera were used to conduct the correlation analysis in the present study. Namely, there were 14 samples for each variable. After running the correlation analysis between paired variables, the Spearman's correlation coefficient was obtained, which ranged from −1.0 to 1.0. Origin 2021 (Correlation Plot app) was used for analysis and visualization.

## Results

### Productive performance and egg quality

The productive performance data during the experimental period of 8 weeks are shown in Table 3. During the feeding period 1 (34–38 weeks of age), the egg production percentage and egg mass in the FPP group were 1.93% and 2.28% higher than those in the CON group, respectively, and the feed intake was 0.15% lower than that in the control group. However, there was no significant difference in any of the three production performance indicators between the two groups (*P* > 0.05). At 38 to 42 weeks of age, the addition of 500 mg/kg FPP to a hen's diet significantly increased the egg production percentage and egg mass by 4.40% and 3.87%, respectively (*P* < 0.05), while the feed intake increased by 0.58%, but there was no statistical significance (*P* > 0.05). During the whole feeding period (34–42 weeks of age), compared with the control group, dietary supplementation with 500 mg/kg of FPP increased the egg production, egg mass, and feed intake of laying hens by 3.11, 3.08, and 0.31%, respectively, and the differences of the first two parameters were statistically significant (*P* < 0.05). From 34 to 38, 38 to 42, and 34 to 42 weeks of age, the FCR of hens fed a diet supplemented with 500 mg/kg FPP decreased by 2.05, 2.58, and 2.33%, respectively, compared to the control group, but none of the differences were detected significantly between the two groups (*P* > 0.05).

**Table 3:** Effects of FPP on the production performance of laying hens.

**Items**	**CON**	**FPP**	**P values**
34–38 weeks of age			
Egg production, %	70.89 ± 0.82	72.26 ± 0.85	0.246
Egg mass, g/d/hen	31.57 ± 0.37	32.29 ± 0.39	0.179
Feed intake, g/d/hen	90.60 ± 0.26	90.46 ± 0.24	0.696
FCR, g feed/g egg	2.92 ± 0.04	2.86 ± 0.04	0.289
38–42 weeks of age			
Egg production, %	64.94 ± 0.78	67.80 ± 0.91	0.018
Egg mass, g/d/hen	29.42 ± 0.36	30.56 ± 0.41	0.036
Feed intake, g/d/hen	89.61 ± 0.23	90.13 ± 0.20	0.090
FCR, g feed/g egg	3.10 ± 0.04	3.02 ± 0.05	0.205
34–42 weeks of age			
Egg production, %	67.92 ± 0.60	70.03 ± 0.64	0.016
Egg mass, g/d/hen	30.49 ± 0.26	31.43 ± 0.29	0.018
Feed intake, g/d/hen	90.11 ± 0.18	90.39 ± 0.15	0.422
FCR, g feed/g egg	3.01 ± 0.03	2.94 ± 0.03	0.104

Data are presented as mean ± SEM. FCR, the ratio of feed intake to egg mass.

The egg quality data of laying hens fed a diet supplemented with or without 500 mg/kg FPP for 8 weeks are shown in Table 4. Compared with the control group, the albumen height and Haugh unit of laying hens fed the FPP supplementation were increased, and the differences in albumen height and Haugh unit between the two treatment groups were extremely significant (*P* < 0.01) and significant (*P* < 0.05), respectively. However, a basal diet containing 500 mg/kg of FPP had no significant effects on egg weight, egg shape index, eggshell thickness, eggshell strength, yolk color, and yolk index compared with the control group (*P* > 0.05).

**Table 4:** Effects of FPP on the egg quality of laying hens.

**Items**	**CON**	**FPP**	**P values**
Egg weight, kg	46.39 ± 0.55	47.46 ± 0.38	0.118
Egg shape index	1.35 ± 0.00	1.34 ± 0.01	0.160
Eggshell thickness, mm	0.44 ± 0.00	0.45 ± 0.00	0.135
Eggshell strength, kg/cm^2^	4.62 ± 0.09	4.69 ± 0.08	0.553
Albumen height	5.25 ± 0.08	5.56 ± 0.08	0.009
Haugh unit	78.56 ± 0.49	80.95 ± 1.06	0.045
Yolk color	8.87 ± 0.06	8.76 ± 0.08	0.269
Yolk index	33.24 ± 0.30	33.13 ± 0.23	0.772

Data are presented as mean ± SEM.

### Jejunal morphology

**Figure 1:**
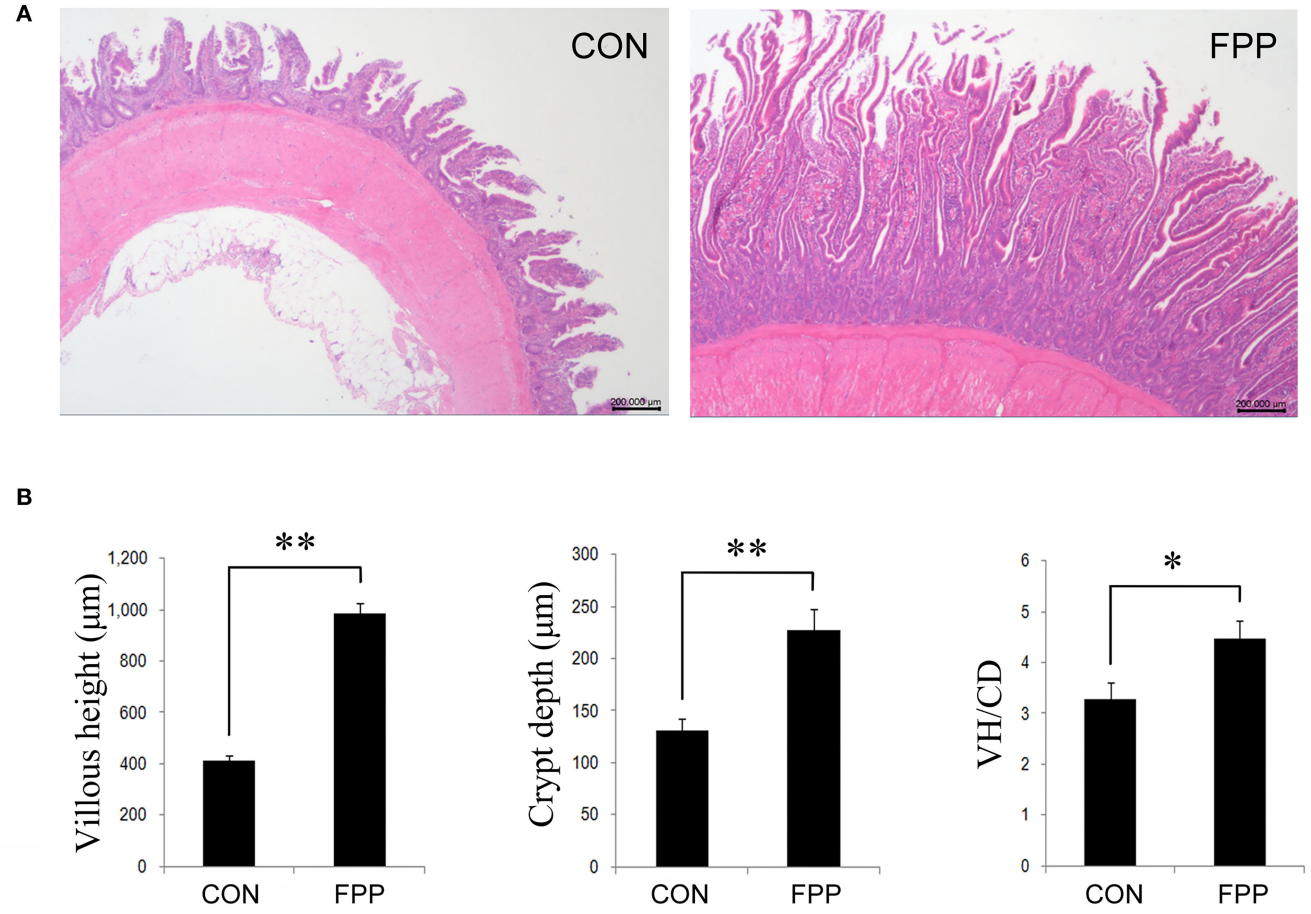
Effect of the fermented plant product (FPP) on jejunal histology and histomorphometry of laying hens. **(A)** Histomorphology of jejunal of laying hens from the CON and FPP treatments. Hematoxylin and erosion (H&E) staining. Scale bar, 200 μm. CON, basal diet without additive; FPP, basal diet + 500 mg/kg of FPP. **(B)** Villus height (VH), crypt depth (CD), and VH/CD ratio in the jejunum of laying hens-fed basal diet with 0 and 500 mg/kg FPP. *P < 0.05, **P < 0.01.

The morphological changes in jejunum of laying hens-fed diets supplementation with 0 or 500 mg/kg FPP for 8 weeks are shown in Figure 1. Compared with the control group, dietary supplementation with 500 mg/kg of FPP significantly increased the villus height (VH) and crypt depth (CD) of jejunum in laying hens (*P* < 0.01). Then the jejunal VH/CD values were calculated, and the result showed that the VH/CD ratio in the FPP group was higher than that in the CON group (*P* < 0.05).

### Jejunal gene expression

**Figure 2:**
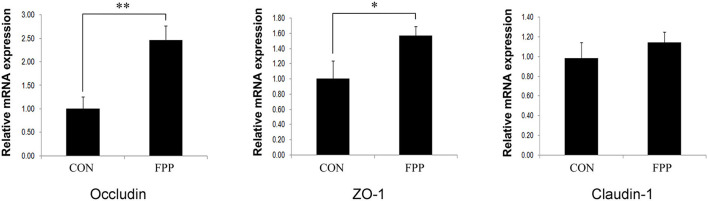
Relative abundance of tight junction proteins *Occludin, ZO-1*, and *Claudin-1* mRNA in the jejunum of laying hens-fed basal diet supplemented with 0 (CON) and 500 mg/kg of the fermented plant product (FPP). β-actin was selected as an internal control. Data are shown as means and standard error (*n* = 8). *P < 0.05, **P < 0.01.

To further investigate the effect of FPP on intestinal barrier integrity, the relative expression levels of tight junction genes *Occludin, ZO-1*, and *Claudin-1* in jejunum were measured. The results of the relative abundance of jejunal *Occludin, ZO-1*, and *Claudin-1* genes in hens-fed diets supplemented with 0 and 500 mg/kg FPP are shown in Figure 2. The mRNA expression levels of *Occludin* and *ZO-1* in the FPP group were higher than those in the CON group, and the expression differences of these two genes between the CON and FPP groups were extremely significant (*P* < 0.01) and significant (*P* < 0.05), respectively. Compared with the control group, 500 mg/kg of FPP added to the hen's diet upregulated the relative expression level of *Claudin-1* mRNA in the jejunum, but the difference was not statistically significant (*P* > 0.05).

### Alpha and beta diversity of cecal microbiota

**Figure 3:**
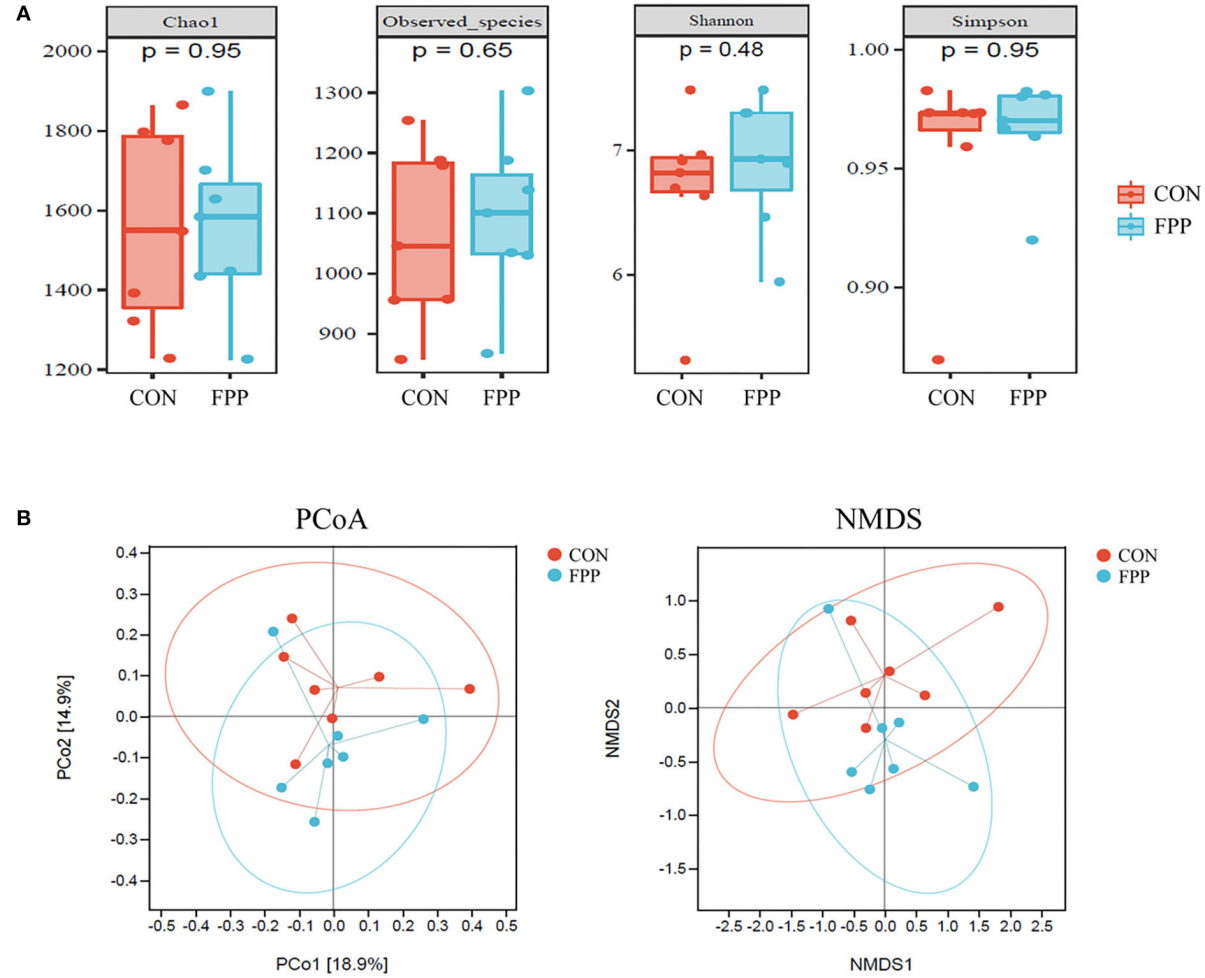
Effect of fermented plant product on the bacterial community diversity of cecal microbiota in laying hens. **(A)** Alpha diversity of cecal bacteria in laying hens-fed basal diet supplemented with 0 (CON) and 500 mg/kg fermented plant product (FPP). Box plots indicate microbiome diversity differences of Chao1 diversity, observed species, Shannon diversity, and Simpson diversity in CON and FPP groups. **(B)** Beta diversity analysis of cecal microbiota in the CON and FPP groups. PCoA and NMDS based on Bray–Curtis distance were applied to identify the separation of the samples. Red and blue dots represent samples collected from laying hens in the CON group and the FPP group, respectively.

To evaluate the species richness and diversity of the cecal microbiota of laying hens from the control and FPP groups, the alpha diversity values (Chao1, observed species, Shannon, and Simpson) were analyzed based on 16S rRNA gene sequencing data. The results of alpha diversity analysis showed that layers fed with 500 mg/kg of FPP had higher Chao1, observed species, and Shannon indices than those in the control group, while the Simpson index of cecal microflora from the control group was slightly higher than that of the FPP group. However, there was no significant statistical difference in alpha diversity values between these two groups (Figure 3A).

To visualize the overall similarities and differences of the samples, principal coordinate analysis (PCoA) and (non-metric multidimensional scaling) NMDS plots derived from Bray–Curtis distance were generated (Figure 3B). The PCoA result revealed that the cecal microbiota of the FPP group was not well separated from that of the control group. The NMDS plot ordinations showed that the cecal microbial communities were separately clustered in the control and FPP groups, but the clustering of the two groups was not so distinct.

### FPP changes the bacterial community composition of cecal microbiota

**Figure 4:**
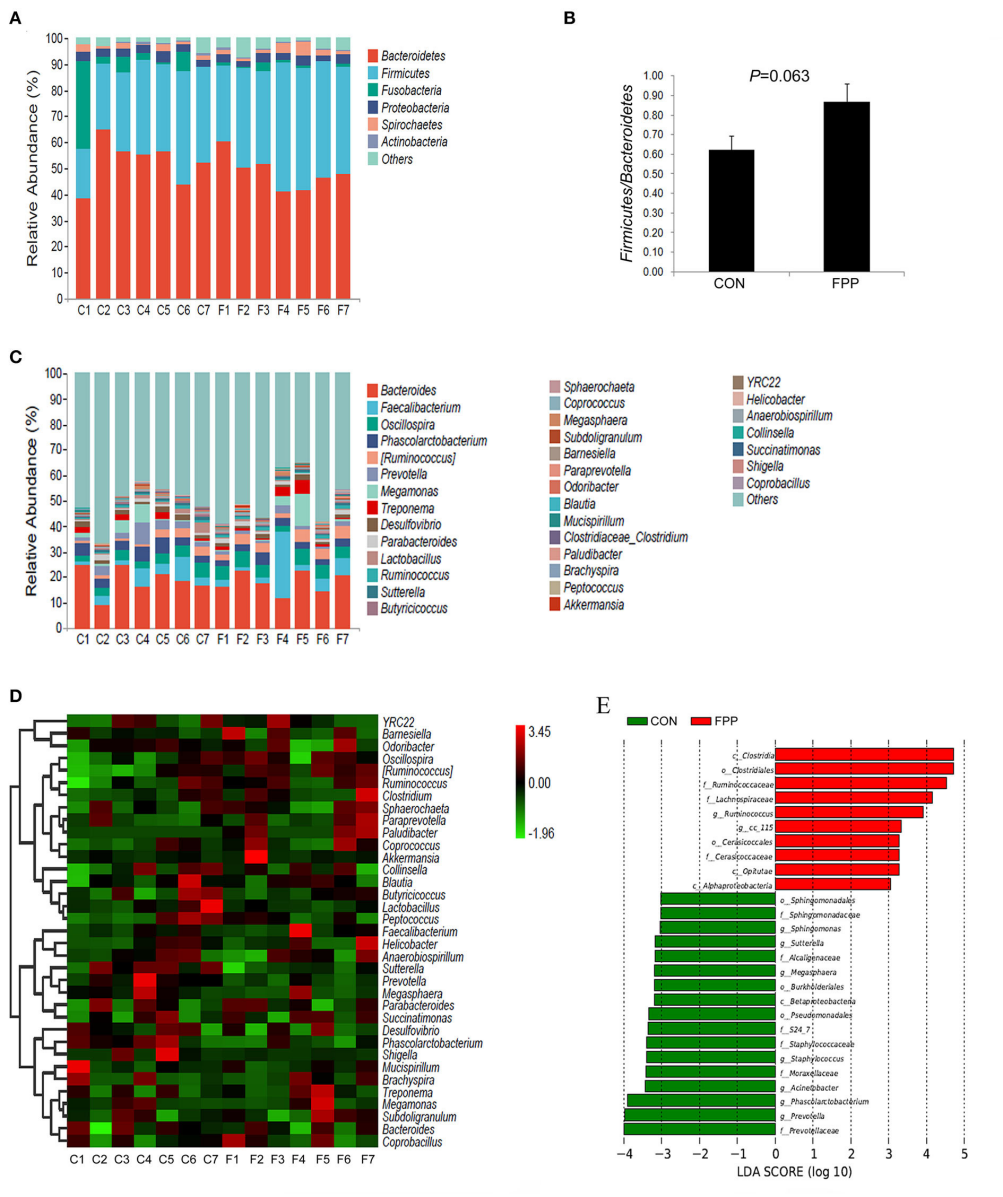
Changes in the cecal microbial community in response to the addition of fermented plant product. Community taxonomic composition and relative abundance of cecal microbiota in individual birds from CON (C1–C7) and FPP (F1–F7) groups at phylum **(A)** and genus **(C)** levels. Ratio of *Firmicutes* to *Bacteroidetes* in each group based on their relative abundance **(B)**. Heatmap of bacterial communities in the CON (C1–C7) and FPP (F1–F7) samples based on the top 35 dominant genera. Rows and columns represent the samples and dominant genera, respectively **(D)**. Linear discriminant analysis (LDA) (LDA>2.0, *P* < 0.05) of cecal microbiomes **(E)**. Histogram of LDA value distribution indicating significant bacterial differences between the CON group (green) and FPP group (red). The prefixes “c_,” “o_,” “f_,” and “g_” represent the annotated level of class, order, family, and genus. CON, basal control diet without additive; FPP, basal diet + 500 mg/kg ferment plant product.

To understand the changes in intestinal bacteria abundance in the samples, the cecal microbiota composition of hens-fed basal diet with or without FPP supplementation was calculated at phylum and genus levels, respectively, and the bar charts representing the relative abundance of cecal bacteria in each sample were further constructed. Based on the relative abundances of bacteria, a column-accumulated graph was generated to visualize the levels and proportions of different phyla in each sample (Figure 4A). The microbial communities were dominated by *Bacteroidetes, Firmicutes, Fusobacteria, Proteobacteria*, and *Spirochaetes*, which accounted for 97.24% of the CON group and 95.90% of the FPP group, respectively. According to the control group, most of the sequences were assigned to *Bacteroidetes* (52.60%), followed by *Firmicutes, Fusobacteria, Proteobacteria*, and *Spirochaetes*, with relative abundances of 32.09, 7.59, 3.24, and 1.72%, respectively. In the FPP group, *Bacteroidetes* (48.57%) also showed overwhelming dominance, followed by *Firmicutes* (40.72%), *Proteobacteria* (2.98%), *Spirochaetes* (2.40%), and *Fusobacteria* (1.23%). FPP tended to increase the relative abundances of *Firmicutes* and *Spirochaetes*, while the abundances of *Bacteroidetes, Fusobacteria*, and *Proteobacteria* were decreased in the cecal microbiota of layers fed 500 mg/kg of FPP. As shown in Figure 4B, dietary FPP supplementation increased the ratio of *Firmicutes* to *Bacteroidetes* compared with the CON group (*P*>0.05). Species composition differences at the genus level are shown in Figure 4C. The predominant genera in the cecal contents of the CON group were *Bacteroides* (19.05%), *Phascolarctobacterium* (4.43%), *Faecalibacterium* (4.17%), and *Oscillospira* (3.83%). The genera *Bacteroides* and *Phascolarctobacterium* were less abundant in the FPP layers, which accounted for 18.17% and 2.96%, respectively. However, the relative abundance of *Faecalibacterium* and *Oscillospira* was higher in the FPP group (6.72 and 4.89%, respectively).

The heatmap analysis of cecal bacterial abundance at the genus level showed that *Clostridium, Succinatimonas, Barnesiella, Ruminococcus, Coprococcus*, and *Faecalibacterium* were more abundant in the FPP group than in the control group, while the birds fed the basal diet had higher abundances of *YRC22, Odoribacter, Lactobacillus, Desulfovibrio, Mucispirillum*, and *Peptococcus* than those fed the diet containing 500 mg/kg FPP (Figure 4D). Moreover, we employed LEfSe to identify bacterial taxa that significantly differentiated between these two groups, and the result is shown in Figure 4E, which showed that 27 discriminative features were identified (LDA score >2, *P* < 0.05). In the CON group, 17 bacterial taxa including *Prevotellaceae* (family), *Prevotella* (genus), *Phascolarctobacterium* (genus), *Acinetobacter* (genus), and *Moraxellaceae* (family) were identified as potential biomarkers. Compared with the CON group, the cecal microbiota structure of birds in the FPP group showed a diverse change, and there were 10 microbial taxa with distinct relative abundances, such as *Clostridia* (class), *Clostridiales* (order), *Ruminococcaceae* (family), *Lachnospiraceae* (family), and *Ruminococcus* (genus).

### Correlation analysis among the tight junction genes and gut microbiota

**Figure 5:**
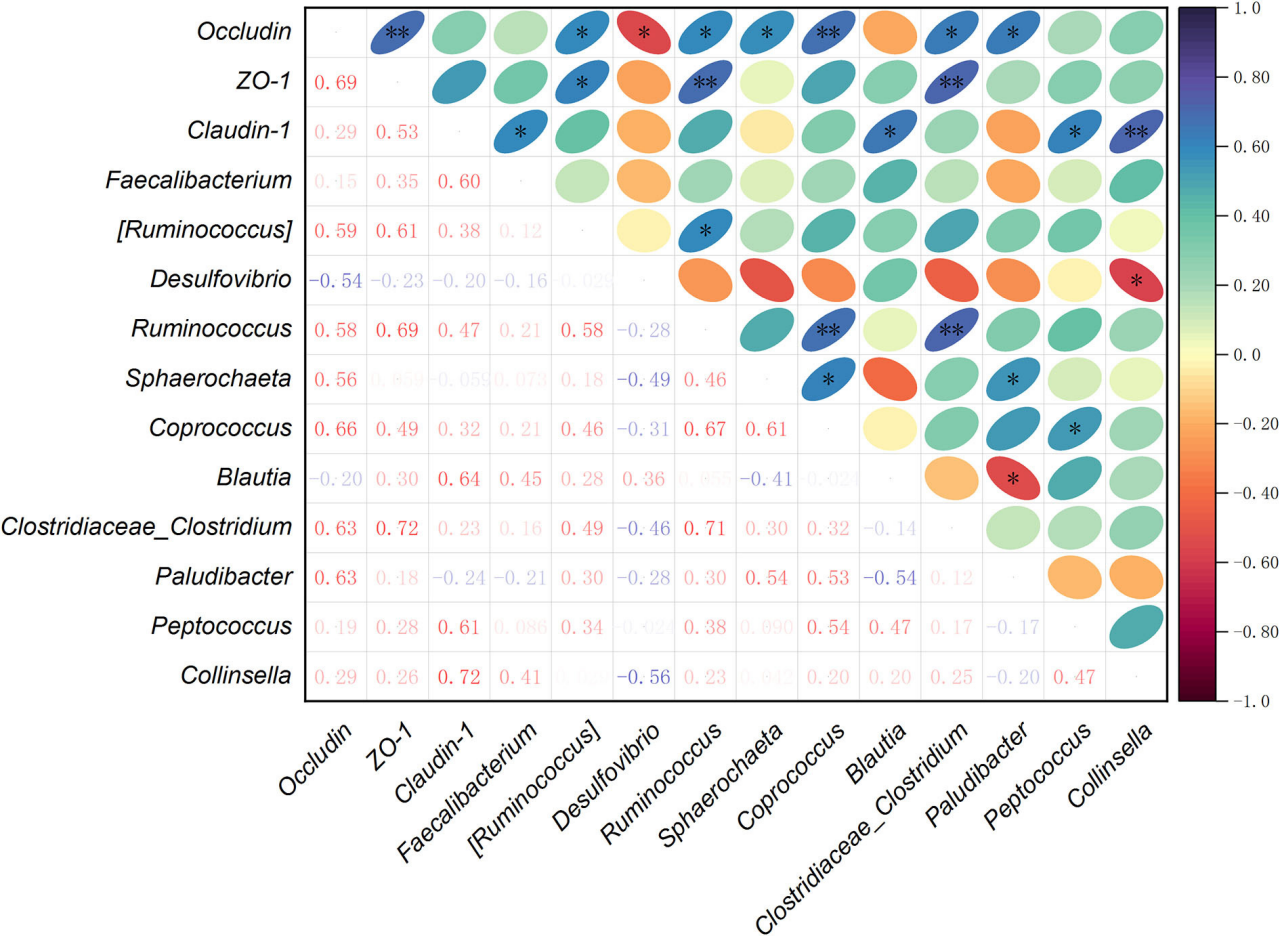
Spearman's correlation analysis among the mRNA expression levels of intestinal barrier-related genes and relative abundances of cecal bacteria selected from the top 35 genera. *P < 0.05, ** *P* < 0.01. The depth of colors ranging from red-purple to navy-blue represents the magnitude of correlation. Significant correlations are marked by *P < 0.05 and **P < 0.01.

To further understand the correlations among the cecal microbiota and intestinal barrier-related genes, Spearman's correlation analysis was carried out to determine their relationships. Based on the mRNA expression levels of *Occludin, ZO-1*, and *Claudin-1* in jejunum, and the relative abundance of main species selected from the top 35 bacterial genera, the correlations among tight junction genes and cecal microbiota were analyzed (Figure 5). The results revealed that the mRNA expression of *Occludin* was positively correlated with the relative abundances of (*Ruminococcus*), *Ruminococcus, Sphaerochaeta, Coprococcus, Clostridiaceae_Clostridium*, and *Paludibacter* (*P* < 0.05), and negatively with *Desulfovibrio* (*P* < 0.05). *ZO-1* mRNA expression showed a positive correlation with (*Ruminococcus*), *Ruminococcus*, and *Clostridiaceae_Clostridium* (*P* < 0.05). The mRNA expression of *Claudin-1* was positively correlated with *Faecalibacterium, Blautia, Peptococcus*, and *Collinsella* (*P* < 0.05). In the relationships between the tight junction genes, there was a positive correlation between *Occludin* and *ZO-1* mRNA expression levels (*P* < 0.05). Among the microbial genera, *Ruminococcus* had a positive correlation with (*Ruminococcus*), *Coprococcus*, or *Clostridiaceae_Clostridium* (*P* < 0.05). The abundance of *Sphaerochaeta* was positively correlated with *Coprococcus* and *Paludibacter* (*P* < 0.05). Moreover, *Coprococcus* had a positive correlation with *Peptococcus* (*P* < 0.05), while *Paludibacter* showed a negative correlation with *Blautia* (*P* < 0.05). The relative abundance of *Desulfovibrio* was negatively correlated with *Collinsella* (*P* < 0.05).

## Discussion

Fermented plant product is rich in nutrients and bioactive substances, which have antibacterial and antioxidant effects, but also display certain preventive and therapeutic activities against cardiovascular diseases, obesity, and other diseases. Laying production and egg quality are the main parameters to assess the performance of laying hens, and improving the layer's performance is one of the key measures to obtaining good economic benefits. In this study, dietary supplementation with 500 mg/kg FPP significantly increased egg production and egg mass of hens during 38–42 weeks of age, and similar results were found throughout the feeding period. Moreover, compared with the basal diet, the addition of 500 mg/kg FPP significantly improved albumen height and Haugh unit. Our findings were consistent with previous reports, which showed that dietary supplementation with plant fermentation products was able to affect laying performance and egg quality of layers. Diets supplemented with 1 and 2% fermented phytogenic feed additive could improve laying performance, egg weight, albumen height, and Haugh unit of layers (Park et al., 2016). Yoshida et al. (2017) evaluated the effect of the fermented spent mushroom substrate as a dietary additive on egg productivity, suggesting that increased egg production might be related to the protection against stress through the fermentation of waste mushroom beds. Fermented *Astragalus* used as an efficient dietary additive could significantly promote the production performance of laying hens during the late laying period (Shi et al., 2020). A recent study demonstrated that the dietary supplementation of fermented pine needle extract (FPNE) significantly improved egg production percentage and egg mass (Kothari et al., 2021). Notably, these reports also indicated that dietary supplementation with plant fermentation products affected FCR and (or) eggshell strength, but these results have not been found in our current study. In addition, the supplementation of FPNE in the layer's diet resulted in a decrease in the Haugh unit, which was contrary to our findings. These differences with this report might be related to the different fermentation substrates, strains, and conditions, as well as the extraction process of effective active substances in fermentation products. The main components of FPP used in this experiment contained *Bacillus subtilis, Lactobacillus acidophilus*, and bioactive substances such as polysaccharides, flavonoids, polyphenols, phenolic acids, fatty acids and esters, sterols, alkaloids, lignans, anthocyanins, and curcumin, which are known to play important roles in regulating intestinal microbiota, antibacterial, anti-inflammatory, antioxidant, and immune modulation. This study illustrated that the additive in the diet improved egg production percentage and egg mass of laying hens, which may be attributed to the improvement of body health by probiotics and bioactive compounds in FPP.

It is generally considered that intestinal mucosa can prevent the passage of harmful substances from the lumen, resist the invasion of pathogenic bacteria, and play a key role in the digestion and absorption of nutrients. VH, CD, and VH/CD are important histomorphological indicators to evaluate the intestinal absorption capacity of animals, and an increase in the VH/CD ratio is always considered favorable for absorption. Lokaewmanee et al. (2012) reported that the villus height in the duodenum and jejunum of broilers-fed diets supplemented with fermented plant products tended to increase. Diets including 1% fermented sour cherry kernel increased the ratio of VH to CD (Gungor and Erener, 2020). The duodenal and jejunal VH were increased in broilers-fed diets supplementation with fermented *Ginkgo biloba* leaf, while the jejunal CD was decreased (Zhang et al., 2015). In the duodenum, jejunum, and ileum of birds, the VH and ratio of VH to CD increased with the increment of the fermentation concentrate of *Hericium caput-medusae* (Bull.:Fr.) Pers. levels (Shang et al., 2016). Compared to the wheat bran and control groups, intestinal morphology of broilers showed that a 5% fermented product supplementation significantly increased the VH in the ileum and jejunum (Lin and Lee, 2021). Similarly, Ding et al. (2020) also reported that fermented tea residue added to the diets of pigs increased the VH and VH/CD ratio of jejunum. Piglets-fed fermented soybean meal had a significant increase in the VH and VH/CD ratio in duodenum, jejunum, and ileum, and a decrease in the CD, compared to those fed with soybean meals alone (Zhu et al., 2017). Consistent with the above reports, in the current study, the supplementation of 500 mg/kg FPP significantly increased the VH and VH/CD ratio in jejunum compared with the control group. As jejunum is an important organ for nutrient absorption, an improvement in VH and VH to CD ratio indicates better nutrient absorption. It is suggested that the higher laying performance of FPP-fed layers could be possibly attributed to the improvements in jejunal morphology. Nevertheless, we also found an increase in the jejunal CD in FPP-fed hens, which might be related to one or more components of plant fermentation products, which needs further experimental verification.

Intestinal epithelial cells serve as an important physical barrier against external environment and stimuli, such as pathogens and toxins, and their integrity is maintained by a series of intercellular junction complexes including tight junctions (Schneeberger and Lynch, 2004). Tight junction proteins are known to have a crucial role in maintaining the integrity of the intestinal epithelial barrier, and their loss is closely related to the pathophysiology of various gastrointestinal disorders (Parikh et al., 2019). Previous studies have indicated that fermented phytogenic feed additive is able to reinforce intestinal barrier integrity through increased gene expression in tight junction signaling. Cheng et al. (2020) discovered that fermented blueberry pomace supplementation increased the expression of *ZO-1, Claudin-1*, and *Occludin* in the ileum of high-fat diet-fed mice. Similar results were reported by Lin and Lee (2021), who found that *Laetiporus sulphureus* fermented product supplementation significantly improved the expression of these three tight junction genes in the jejunum of broiler chickens. These results were consistent with our findings, which showed that FPP supplementation increased the mRNA expression levels of *Occludin, ZO-1*, and *Claudin-1* genes in the jejunum of laying hens and that the differences in the expression of *Occludin* and *ZO-1* were statistically significant. Occludin is an important tight junction protein that contributes to the stability of cell-to-cell tight junctions and optimal barrier properties. Increased expression of *Occludin* mRNA improved intestinal barrier function and its defense status (Zhang et al., 2020). ZO-1, a cytoskeletal protein, is a member of the zona occludens family, which is an essential part of the tight junctions in cytoplasmic plaques and plays a crucial role in regulating intestinal permeability and integrity (Gilani et al., 2018). A previous research has shown that increased mRNA expression of intestinal barrier gene *ZO-1* could be related to the inhibition of pro-inflammatory cytokine expression, and consequently caused the improvement of intestinal epithelial barrier function in laying hens (Feng et al., 2021). Claudin-1 is one of the most important components of the intestinal epithelial barrier, which can strengthen the stability of the intestinal mucosal mechanical barrier, and plays an important role in maintaining intestinal mucosal permeability and preventing the invasion of harmful substances (Song et al., 2018). The high expression of *Claudin-1* led to increased epithelial cell tightness and decreased solute permeability (Awad et al., 2017). Overall, in this study, the expression levels of tight junction proteins such as *Occludin, ZO-1*, and *Claudin-1* increased with the FPP supplementation, which may enhance intestinal barrier function to some extent.

The gut tract of animals is colonized with a diverse and complex microbiota that plays an important role in maintaining host health and affecting production performance (Diaz Carrasco et al., 2019; Khan et al., 2020). Until recently, research on the intestinal microbiota of poultry has mainly relied on traditional microbiology techniques, which can only culture a small portion of the gut microbial community. Next-generation sequencing of the 16S rRNA gene is a powerful tool for studying the biological role of gut microbiota. The diversity of intestinal microbiota of chickens is largely influenced by age, location of the digestive tract, and diet. The richness and diversity of the gut microbiota are usually estimated by the Chao1, observed species, Shannon, and Simpson indices. Among them, the Chao1 and observed species indices are widely used to assess the richness of the ecosystem, and the Shannon and Simpson indices are the most widely accepted measures of ecological diversity. In the present study, dietary supplementation with 500 mg/kg FPP did not alter the α-diversity values of cecal microbiota as evidenced by the similar Chao1, observed species, Shannon, and Simpson indices between different treatments of the laying hens. Furthermore, beta diversity was employed to compare the microbial community composition among different samples. Our results of PCoA and NMDS analysis showed that the microbial community structure of the FPP group was weakly separated from that of the CON group. Thus, additional statistical tests may be more informative than ordinations for identifying FPP-associated taxa.

The composition of the gut microbiota changes with age, breed, diet, and production system of poultry. Most studies related to gut microbiota have been conducted in broilers, which have different microbial communities compared to those of layers. According to the data on gut microbiota in laying hens, *Bacteroidetes* and *Firmicutes* are the dominant bacterial communities at the phylum level (Dong et al., 2017), which is in agreement with our findings. We found that *Bacteroidetes* and *Firmicutes* were the two most dominant phyla in the cecal microbiota of all tested hens, followed by *Fusobacteria* and *Proteobacteria*. Our results demonstrated that feeding diets containing 500 mg/kg FPP additive increased *Firmicutes*, while decreasing *Bacteroidetes* and *Proteobacteria* in the cecum of birds. Likewise, Xie et al. (2017) detected that fresh fermented soybean meal increased *Firmicutes* and reduced *Bacteroidetes* and *Proteobacteria* in the cecum of piglets. An increase in the abundance of phylum *Firmicutes* has been proved to be positively correlated with the energy and nutrient absorption, whereas the increment of phylum *Bacteroidetes* was linked with poor nutrient digestibility (Turnbaugh et al., 2006; Jumpertz et al., 2011). The decrease in phylum *proteobacteria* can be considered beneficial because its increase has been associated with intestinal inflammation (Mukhopadhya et al., 2012), and the bacteria included in this phylum are well known to cause inflammatory bowel disease (IBD) in animals. Therefore, it is suggested that the increased abundance of *Firmicutes* along with the decreased abundance of *Bacteroidetes* and *proteobacteria* might contribute to nutrient utilization and anti-inflammatory status, and ultimately improve the laying performance in hens. In addition, this study showed that the intestinal microecosystem of laying hens fed a diet containing 500 mg/kg of FPP changed at the phylum level by increasing the abundance of *Firmicutes* at expense of *Bacteroidetes*, and subsequently resulting in an increment of *Firmicutes* to *Bacteroidetes* ratio, which is generally regarded as a biomarker of intestinal function and can indicate the state of microecological balance (Cheng et al., 2017). Several studies have suggested that the higher ratio of *Firmicutes* to *Bacteroidetes* may play an important role in energy uptake and ultimately lead to weight gain (Ley et al., 2006) or improved egg-laying performance (Elokil et al., 2020). We hypothesized the improvement of the laying performance with FPP added to the hen's diet may be attributed to the increased abundance of *Firmicutes* and *Firmicutes* to *Bacteroidetes* ratio in the cecal microbiota of laying hens, which is beneficial to the utilization of nutrients by animals.

At the genus level, the cecal microbiota of birds from CON and FPP groups was mainly dominated by *Bacteroides, Faecalibacterium, Oscillospira*, and *Phascolarctobacterium*. It seemed that dietary containing 500 mg/kg FPP increased the relative abundance of *Faecalibacterium* and *Oscillospira* by 60.92 and 27.68%, respectively, whereas FPP supplementation reduced the relative abundance of *Bacteroides* and *Phascolarctobacterium* by 4.66 and 33.19%, respectively, which could lead to the conclusion that the addition of FPP had very little effect on the abundance of *Bacteroides*. As the major short-chain fatty acid producers, *Faecalibacterium* provides energy sources for intestinal epithelial cells by metabolizing dietary fibers, thus exerting anti-inflammatory effect (Hiippala et al., 2018). Wang et al. (2020) demonstrated that the increased *Faecalibacterium* may regulate the immunity of breeding geese to improve the laying performance. For the genus *Oscillospira*, some species may utilize host glycans and probably produce short-chain fatty acids, among which butyrate plays an important role in preventing inflammation by inducing T cell differentiation (Furusawa et al., 2013) and regulating the expression of genes encoding pro-inflammatory cytokines (Chang et al., 2014). However, *Phascolarctobacterium* were detected to be higher in patients with multiple diseases such as major depressive disorder (Cheung et al., 2019) and autism spectrum disorder (Iglesias-Vázquez et al., 2020).

In the present study, heat map analysis of the top 35 bacterial genera indicated that the microbiota composition and abundance in the FPP group were different from those in the CON group. The addition of FPP increased the relative abundance of *Clostridium* and *Ruminococcus*, producers of short-chain fatty acids, which were found to be inversely associated with the severity of IBD (Franzosa et al., 2019; Lavelle and Sokol, 2020). *Ruminococcus* was also reported to be related to polysaccharide degradation and utilization in chickens (Nguyen et al., 2021). These two bacteria produce short-chain fatty acids, which may be the main source of energy for intestinal epithelial cells, create an environment for appropriate intestinal colonization, and exhibit immunomodulatory and anti-inflammatory properties (Parada Venegas et al., 2019). Moreover, we observed that *Odoribacter* and *Desulfovibrio* were more abundant in the CON group than in the FPP group, which were reported to be harmful to health. A recent study indicated that compared with healthy subjects, major depressive disorder patients have higher abundance of *Odoribacter*, which may play an important role in the pathogenesis of cognitive impairment in patients with major depression (Liu et al., 2022). For the genus *Desulfovibrio*, it always flourishes in the inflammatory environments. The fermented feed may perform its anti-inflammatory effects by reducing the prevalence of sulfate and minimizing the abundance of *Desulfovibrio* (Sawin et al., 2015). Convincing data obtained from human subjects have shown an increased abundance of *Desulfovibrio* in patients with intestinal diseases (Rowan et al., 2010). Additionally, there were 17 and 10 potential biomarkers with significantly different abundance in the CON and FPP groups, respectively, and further research are needed to explore the functions of these biomarkers in regulating the gut healthy of laying hens. Together, our findings about cecal microbiota demonstrated that diet supplementation with fermented plant products maintained a healthy intestinal ecosystem due to the increase in beneficial bacteria groups in the cecum microbial composition of birds, namely, *Firmicutes, Faecalibacterium, Clostridium*, and *Ruminococcus*, and the decrease in potentially harmful flora such as *Proteobacteria, Phascolarctobacterium, Odoribacter*, and *Desulfovibrio*.

Furthermore, the relationships between the tight junctions and cecal microbiota were analyzed in this study. Here we focus on the roles of cecal bacteria genera in regulating intestinal barrier-related genes. Our result indicated that *Faecalibacterium, Ruminococcus, Coprococcus*, and *Blautia* were positively correlated with the intestinal barrier markers, whereas *Desulfovibrio* had a negative correlation with the expression of *Occludin*. As previous reports described, *Faecalibacterium prausnitzii, Ruminococcus bromii, Ruminococcus torques, Coprococcus eutactus, Coprococcus catus*, and *Blautia obeum* are the major short-chain fatty acids producers, which have been shown to be associated with anti-inflammatory and immunity (Louis and Flint, 2017). *Desulfovibrio* can degrade sulfate and produce hydrogen sulfide, which is regarded as a genotoxic substance that might be involved in colon inflammation, so this genus is always considered to be a pro-inflammatory microflora (Singh and Lin, 2015; Simpson et al., 2021). Therefore, we hypothesized that dietary FPP supplementation may promote the expression of intestinal barrier-related genes by improving the cecal microflora of laying hens. These results provided a theoretical basis for understanding the relationship between the cecal microbiota and intestinal barrier function modulated by FPP, although further investigations on the regulation mechanism of intestinal bacteria on tight junction proteins should be conducted in future.

In summary, our results indicated that the dietary supplementation of 500 mg/kg FPP can promote egg production, egg mass, albumen height, and Haugh unit of laying hens, and FPP as an efficient dietary additive may improve the gut health of birds by enhancing expression levels of tight junctions, ameliorating intestinal morphology and altering microbial community structure of cecum.

## Data availability statement

The datasets presented in this study can be found in online repositories. The names of the repository/repositories and accession number(s) can be found below: https://www.ncbi.nlm.nih.gov/, PRJNA839201.

## Ethics statement

The animal study was reviewed and the animal experimental procedures in this study were carried out in accordance with the national standard of Laboratory animal-guideline for ethical review of animal welfare (China) and approved by Institute of Animal Husbandry and Veterinary Science, Zhejiang Academy of Agricultural Sciences (Hangzhou, China).

## Author contributions

YT participated in the design of the study, analyzed and validated the data, and wrote the manuscript. GL, SZ, TZ, and LC carried out the experiments. ZT provided important technical advice and participated in some experiments. LL conceived this study and contributed to the funding acquisition. All authors have checked, read, and approved the final version of the manuscript.

## Funding

This work was supported by the Key Research and Development Program of Zhejiang Province (No. 2021C02034), Zhejiang Science and Technology Major Program on Agricultural New Variety Breeding (No. 2021C02068), and Zhejiang Provincial Special Commissioner Team Projects of Science & Technology (No. Xianju Chicken Industry, 2020–2024).

## Conflict of interest

The authors declare that the research was conducted in the absence of any commercial or financial relationships that could be construed as a potential conflict of interest.

## Publisher's note

All claims expressed in this article are solely those of the authors and do not necessarily represent those of their affiliated organizations, or those of the publisher, the editors and the reviewers. Any product that may be evaluated in this article, or claim that may be made by its manufacturer, is not guaranteed or endorsed by the publisher.
